# Analgesic effects of *Marasmius androsaceus* mycelia ethanol extract and possible mechanisms in mice

**DOI:** 10.1590/1414-431X20177124

**Published:** 2018-03-01

**Authors:** Jia Song, Xue Wang, Yu Huang, Yidi Qu, Guirong Zhang, Di Wang

**Affiliations:** 1 School of Life Sciences, Jilin University, Changchun, Jilin, China; 2 The College of Biotechnology, Tianjin University of Science and Technology, Tianjin, China; 3 Zhuhai College of Jilin University, Jilin University, Zhuhai, China

**Keywords:** Marasmius androsaceus, Analgesic ability, Monoamine neurotransmitters, Calcium channels

## Abstract

*Marasmius androsaceus* is a medicinal fungus mainly used to treat various forms of pain in China. This study investigated the analgesic effects of an ethanol extract of *M. androsaceus* (MAE) and its potential molecular mechanisms. Oral administration of MAE (50, 200, and 1000 mg/kg) had significant analgesic effects in an acid-induced writhing test, a formalin test, and a hot-plate test, with effectiveness similar to tramadol (the positive control drug). The autonomic activity test showed that MAE had no harmful effects on the central nervous system in mice. MAE resulted in significantly enhanced levels of noradrenalin and 5-hydroxytryptamine in serum but suppressed both of these neurotransmitters in the hypothalamus after 30 s of hot-plate stimulation. Co-administration with nimodipine (10 mg/kg; a Ca^2+^ channel blocker) strongly enhanced the analgesic effect in the hot-plate test compared to MAE alone. Moreover, MAE down-regulated the expression of calmodulin-dependent protein kinase II (CaMKII) in the hypothalamus after a 30-s thermal stimulus. These results suggested that the analgesic ability of MAE is related to the regulation of metabolism by monoamine neurotransmitters and Ca^2+^/CaMKII-mediated signaling, which can potentially aid the development of peripheral neuropathic pain treatments obtained from *M. androsaceus*.

## Introduction

Pain is a physiological and pathological phenomenon and a symptom of various diseases (1), and its management has attracted considerable attention from researchers worldwide (2). Unlike other conditions, pain requires in-depth diagnosis and multiple therapies. The two major theories for the pharmacological mechanism of pain treatment are the central nervous system hypothesis and the peripheral nervous system hypothesis. Opioid receptors, choline receptors, Na^+^/K^+^ ion channels, and Ca^2+^ ion channels are reported to be targets for analgesic drugs (3). The analgesic agents currently used for pain treatment fall into two major classes. Narcotic analgesics provide effective pain relief, but their severe adverse effects, especially addiction, limit their clinical application (4). Alternatively, non-steroidal anti-inflammatory drugs (NSAIDs) are commonly used to moderate pain following injury and minor surgery, and in combination therapy for various diseases (5,6). Unfortunately, long-term administration of NSAIDs can cause aplastic anemia (7), platelet reduction (8), and viscera damage (9).

Herbal medicine has attracted worldwide attention as a versatile source of pharmacological agents with few adverse effects. According to epidemiological studies, 41 to 62% of cancer patients receive auxiliary therapeutics that contain herbal medicine, mostly for pain relief (10,11). Encouragingly, our group has demonstrated that an herbal medicine, the Jia-Yuan-Qing Pill, shows antinociceptive activities via regulation of peripheral nerves and does not induce physical dependence (12). Moreover, through regulation of monoamines, nNOS/K_ATP_ channel, and mAChR pathway, a *Paecilomyces hepiali* extract shows notable antinociceptive activities in a mouse model (13). These findings illustrate the potential for developing novel painkillers from natural products.

*Marasmius androsaceus* (MAE), a medicinal fungus, shows various pharmacodynamic activities, especially for treating pain-related diseases (14). Similarly to the fruit bodies of *M. androsaceus*, the mycelium obtained from submerged fermentation of the species contains a variety of pharmacologically active ingredients, including polysaccharides, alkaloids, cholesteryl acetate, and flavone (15). “An-Luo Tong”, a painkiller widely used in clinics in China, is produced from *M. androsaceus* mycelium. Although *M. androsaceus* has been used as an analgesic for many years, its pharmacological mechanisms have still not been studied in detail.

In this study, we investigated the analgesic effect of an ethanol extract of MAE mycelia and compared this effect with that of tramadol in an acetic acid-induced writhing test, a formalin-induced test, and a hot-plate test.

## Material and Methods

### Preparation of *M. androsaceus* mycelia ethanol extract

Following our previously reported method, the mycelia of MAE were obtained by submerged fermentation (15) and extracted with 10 portions of 95% ethanol at 60°C for 3 h, with the extraction performed twice. The MAE extract was obtained via evaporation and dissolved in a solvent mixture of physiological saline solution, ethanol, and Tween80 (v/v/v=98/1/1) before the experiments.

### Analgesic effects of MAE in mouse models

#### Animal care and drug administration protocol

Eight-week-old Kunming mice (20-22 g; specific pathogen-free grade) were obtained from the Laboratory Animal Center of Jilin University (China). The experimental protocol was approved by the Animal Ethics Committee of Jilin University. The mice were allowed to adapt to their new surroundings for 1 week; they were housed at 23±1°C with a 12/12 h light/dark cycle and fed autoclaved standard chow and water *ad libitum*. Twelve hours before the experiment, the mice were fasted overnight.

**Figure 1.**
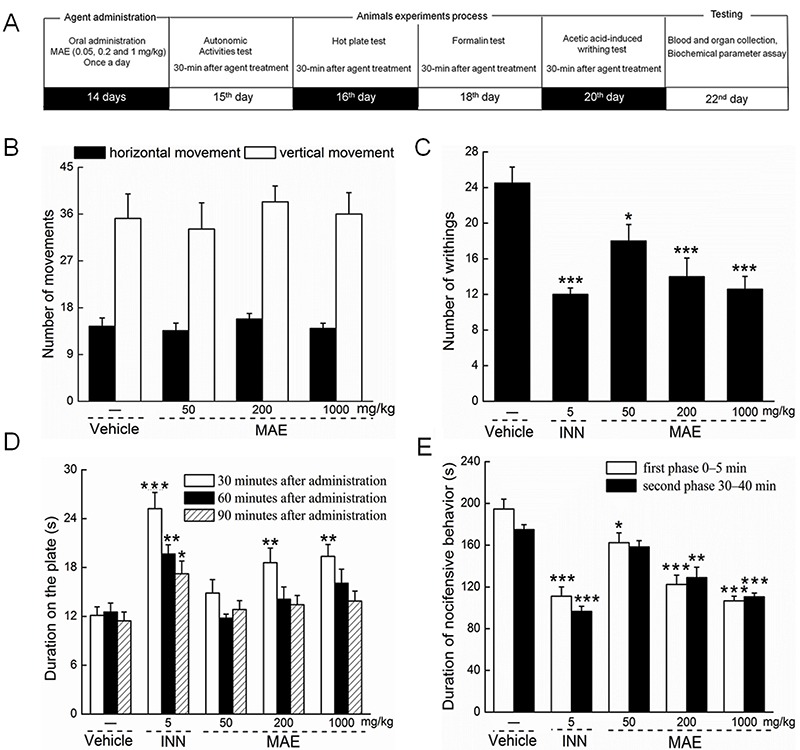
*A*, Experimental protocol and drug administration. Effects of *M. androsaceus* extract (MAE) and tramadol (INN) on (*B*) horizontal/vertical movements, (*C*) acetic acid-induced writhing test, (*D*) hot-plate test, and (*E*) formalin test in BALB/c mice. Data are reported as means±SE (n=10). *P<0.05, **P<0.01, and ***P<0.001 *vs* vehicle group (one-way ANOVA followed by Dunn's test).

Fifty mice were randomly divided into 5 groups (n=10). The control group (CTRL) received sterile saline solution at 2.0 mL/kg. The tramadol group received tramadol (INN; Grunenthal GmbH, China; positive drug control) at 5 mg/kg. The MAE groups received MAE at doses of 50, 200, and 1000 mg/kg. All treatments were administered orally for 21 days. After 14 days of treatment, the following experiments were performed. The detailed experimental protocol and agent administration are shown in Figure 1A.

#### Autonomic activity test

On the 15th day, 30 min after drug treatment, the mice were placed in a multifunctional mice independent activities recorder (ZZ-6; Chengdu Techman Software Co., Ltd., China) and allowed to adapt to the environment for 2 min, with the chamber covered by a light-blocking plate. The number of autonomic activities performed by the mice over 5 min, including horizontal movements and vertical movements, was recorded.

#### Hot-plate test

On the 16th day, 30 min after drug treatment, a hot-plate test was performed (16). The mice were placed on a hot plate at a temperature of 55±0.5°C. The latency to the first sign of hind paw licking or jumping to avoid thermal nociception was taken as an index of the nociceptive threshold. The nociceptive threshold was measured at 30, 60, and 90 min after the last oral administration.

#### Formalin test

On the 18th day, 30 min after drug treatment, a formalin test was performed. Five minutes before the experiment, the mice were placed in a 20-cm diameter transparent beaker to adapt to the environment. Following the method of previous research (17), 20 μL of 2% formalin was injected into the dorsal surface of the right hind paw of each mouse. The number of times that the mice licked the affected area between 0 and 5 min and between 30 and 35 min after injection was recorded.

#### Acetic acid-induced writhing test

On the 20th day, 30 min after drug treatment, an acetic acid-induced writhing test was performed following the method of previous studies (18). The mice received intraperitoneal injections of 0.2 mL 0.6% acetic acid, and their writhing responses were recorded over 20 min.

#### Measurement of biochemical parameters

On the 22nd day, before the 30-s thermal stimulus test, blood was collected from the caudal vein of each mouse. After 1 h of relaxation, the mice were subjected to 30-s thermal stimulus on the hot plate at a temperature of 55±0.5°C. The mice were immediately decapitated, and blood and hypothalamus tissues were collected. An enzyme-linked immunosorbent assay kit (Shanghai Yuanye Bio-Technology Co., Ltd, China) was used to determine the levels of norepinephrine (NE) and 5-hydroxytryptophan (5-HT) in the serum and hypothalamus tissues before and after thermal stimulation.

### Investigation of mechanism

One hundred and forty Kunming mice (8 weeks old, 20 to 22 g, specific pathogen-free grade) were obtained from the Laboratory Animal Center of Jilin University (China) and cared for under the same protocol as above. The mice were randomly separated into 14 groups (n=10).

Six inhibitors/blockers (Sigma-Aldrich, USA) were administered to investigate the possible mechanism of MAE. Normal mice treated with 0.2 mL deuterium-depleted water served as the control group. Naloxone (1 mg/kg; non-selective opioid receptor antagonist), 10 mg/kg methylene blue (guanylate cyclase inhibitor), 2 mg/kg yohimbine (α2 adrenergic antagonist), 2 mg/kg glibenclamide (ATP-sensitive K^+^ channel antagonist), 5 mg/kg atropine (M-acetylcholine receptor antagonist), and 10 mg/kg nimodipine (Ca^2+^ channel blocker) were administered to the mice 15 min before oral administration of 200 mg/kg MAE, and continued for 14 days. Thirty min after the last MAE treatment, the hot-plate test was performed, and the paw withdrawal latency was recorded.

### Western blotting

The hypothalami collected from the mice were lyzed using radioimmunoprecipitation assay buffer (Sigma-Aldrich) containing 1% protease inhibitor cocktail. The concentration of total protein was determined by a bicinchoninic acid protein assay kit (Beyotime, China). Forty micrograms of protein was separated by 10% SDS-PAGE and transferred onto a 0.45 m PVDF membrane (Bio Basic, Inc., USA). The membranes were blocked with 5% bovine serum albumin for 4 h, then probed with primary antibody phosphor (P)-Ca^2+^/calmodulin-dependent protein kinase II (CaMKII), total (T)-CaMKII, and glyceraldehyde-3-phosphate dehydrogenase (Santa Cruz, USA) at 4°C overnight, then washed three times in phosphate-buffered saline solution for 15 min. The secondary antibodies (1:2000) were incubated at room temperature for 4 h. The membranes were washed again three times in phosphate-buffered saline solution. The bands were visualized with an ECL Advance kit for 3 to 5 min and quantified using ImageJ software (National Institutes of Health, USA).

### Statistical analysis

All data were reported as mean±SE. Differences were determined by one-way analysis of variance followed by *post hoc* multiple comparisons (Dunn's test) using SPSS 16.0 software (IBM Corporation, USA). Statistical significance was declared for P values of less than 0.05.

## Results

### Analgesic effects of MAE

MAE had no significant effect on the regulation of horizontal and vertical movement in the autonomic activity test, indicating its safety and non-toxicity to the central nervous system in mice (P>0.05; Figure 1B). The acetic acid-induced writhing test is a widely accepted method to measure the extent of analgesic activity (19). Similar to 5 mg/kg INN, MAE significantly reduced body writhing instances compared to the control group, especially at 200 mg/kg [P*<*0.001, *F(1,34)=*14.68] and 1000 mg/kg [P*<*0.001, *F(1,34)=*27.13 of MAE (Figure 1C)].

In the hot-plate test, MAE extended the latency period of mice on the hot plate compared to the control group, but only at 30 min after administration [P*<*0.01, *F(1,12)=*9.56 (MAE at 200 mg/kg); *F(1,11)=*16.68 (MAE at 1000 mg/kg); Figure 1D]. In contrast, INN at 5 mg/kg significantly enhanced the latency period of the mice at 30, 60, and 90 min after administration [P*<*0.05, *F(1,12)=*33.78 (30 min), *F(1,12)=*14.71 (60 min), *F(1,12)=*9.22 (90 min); Figure 1D].

The formalin test is used to distinguish the analgesic effect on the central nervous system from that on the peripheral nervous system (20). MAE significantly reduced the first-phase and second-phase pain responses in formalin-treated mice [P*<*0.05, *F(1,12)=*5.90 (MAE at 50 mg/kg first-phase), *F(1,12)*=31.17 (MAE at 200 mg/kg first-phase), *F(1,12)=*64.36 (MAE at 1000 mg/kg first-phase); *F(1,12)*=17.60 (MAE at 200 mg/kg second-phase), *F(1,11)=*116.06 (MAE at 1000 mg/kg second-phase); Figure 1E], in a manner similar to INN, implying that MAE-mediated antinociceptive effects occur via the central nervous system.

### Effects of MAE on monoaminergic neurotransmitter levels

**Figure 2.**
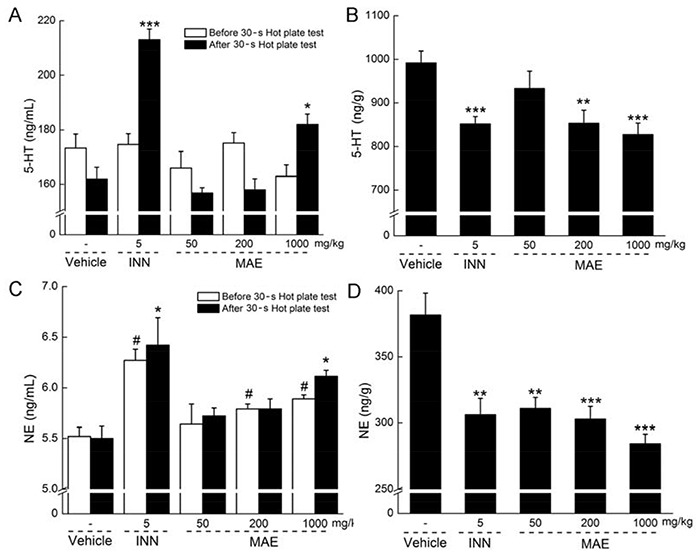
Mice were treated with *M. androsaceus* extract (MAE) (50, 200, and 1000 mg/kg) and tramadol (INN, 5 mg/kg) for 2 weeks. Before and after 30-s thermal stimulation, blood samples and hypothalamus tissues were collected, and the levels of 5-HT and norepinephrine (NE) in the serum (*A, C*) and hypothalamus (*B, D*) were analyzed using ELISA. Data are reported as means±SE (n=10). ^#^P<0.05 and ^###^P<0.001 *vs* control group (before 30-s thermal stimulation); *P<0.05, **P<0.01, and ***P<0.001 *vs* vehicle group (after 30-s thermal stimulation) (one-way ANOVA followed by Dunn's test).

After a 30-s thermal stimulus, 5 mg/kg INN and 1000 mg/kg MAE significantly increased the levels of 5-HT [P*<*0.05; *F(1,11)=*84.36 and *F(1,10)=*12.18; Figure 2A] and NE [P*<*0.05, *F(1,10)*=7.18 and *F(1,10)=*14.35; Figure 2C] in serum compared to the control group. INN (5 mg/kg) and 200 and 1000 mg/kg MAE significantly reduced the 5-HT levels [P<0.01, *F(1,18)=*17.17, and *F(1,18)* =10.59 and 17.49; Figure 2B] and NE levels [P<0.01, *F(1,21)* =13.90, and *F(1,20)=*16.63 and 28.51; Figure 2D] in the hypothalami of the mice after a 30-s thermal stimulus.

### Possible mechanisms of MAE-mediated analgesic activity

**Figure 3.**
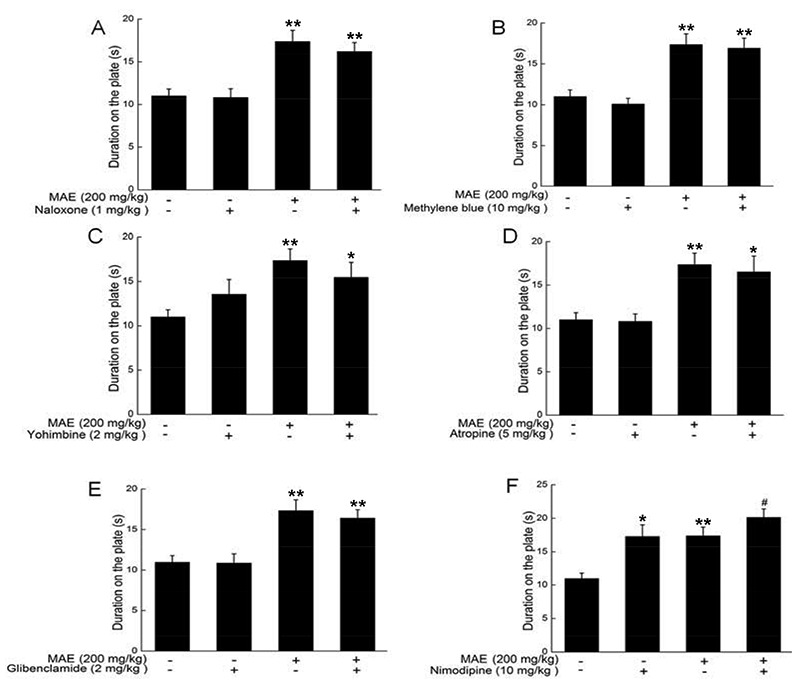
Mice were pre-treated with *A*, 1 mg/kg naloxone, *B*, 10 mg/kg methylene blue, *C*, 2 mg/kg yohimbine, *D*, 5 mg/kg atropine, *E*, 2 mg/kg glibenclamide, and *F*, 10 mg/kg nimodipine via intraperitoneal injection, followed by oral treatment with 200 mg/kg *M. androsaceus* extract (MAE) or deuterium-depleted water. After 30 min, a hot-plate test was performed and the paw withdrawal latency was recorded. Data are reported as means±SE (n=10). *P<0.05 and **P<0.01 *vs* vehicle group. ^#^P<0.05 *vs* MAE only treated mice (one-way ANOVA followed by Dunn's test).

Naloxone (1 mg/kg), 10 mg/kg methylene blue, 2 mg/kg yohimbine, 5 mg/kg atropine, and 2 mg/kg glibenclamide showed no significant effect on MAE-mediated hypo-irritation in the hot-plate test after co-treatment with 200 mg/kg MAE (P>0.05; Figure 3A-E). Co-administration of 10 mg/kg nimodipine and MAE significantly increased the latency period of mice on the hot plate compared to treatment with nimodipine or MAE alone (P*<*0.05, *F(1,15)=*36.12; Figure 3F). Therefore, we speculated that MAE may have pharmacological effects similar to nimodipine or display synergistic activities with nimodipine.

### Effects of MAE on CaMKII activation

**Figure 4.**
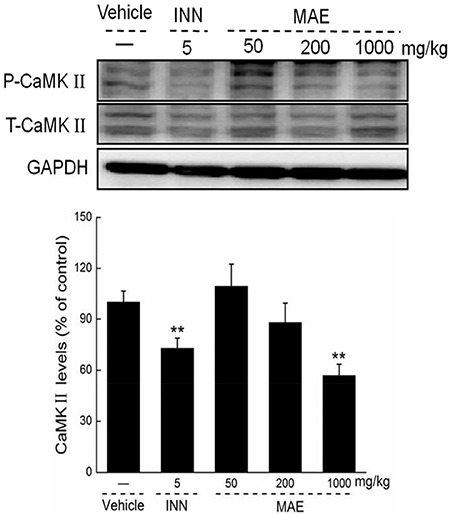
Mice were treated with *M. androsaceus* extract (MAE, 50, 200, and 1000 mg/kg) and tramadol (INN) (5 mg/kg) for 2 weeks. After 30-s thermal stimulation, the activation of CaMKII in the hypothalamus was detected via western blot. Data are reported as means±SE (n=10). **P<0.01 *vs* vehicle group (one-way ANOVA followed by Dunn's test).

Compared to the control group, INN and MAE (1000 mg/kg) strongly inhibited the expression of phosphor-CaMKII in the hypothalamus (P*<*0.01, *F(1,4)=*22.32 and 52.19; Figure 4).

## Discussion

Although the commonly used analgesics are clinically effective, their adverse effects, including allergic reactions and addiction, limit their application. Therefore, the search for new analgesics with fewer side effects has become a major trend. In this study, using the acetic acid-induced writhing test, formalin test, and hot-plate test, we confirmed that MAE showed analgesic activities through the central nervous system. The acetic acid-induced writhing test is a nonselective analgesia model that is suitable for initial screening of drug efficacy (21). Combined with the writhing test, the hot-plate response can be used to distinguish central from peripheral antinociceptive effects (22). In addition, the first phase of the formalin test reflects the direct effect of formalin on nociceptors (non-inflammatory pain), whereas the second phase reflects inflammation (23). Unlike peripheral analgesic drugs, such as aspirin, MAE not only prolonged the reaction time in the hot-plate test, but also provided pain relief in both phases of the formalin test.

In our experiments, MAE only prolonged the reaction time in the hot-plate test after 30-min administration. This phenomenon may be related to the *in vivo* metabolism of MAE and its possible working pathway (Ca^2+^/CaMKII signaling). More experiments focused on metabolism of MAE will be performed in our group. Additionally, the MAE-mediated analgesic effects in mice were found to be non-dose-dependent. As a medicinal fungus, crude *M. androsaceus* may contain multiple effective components that “systemically target” the pain response in a natural way. This mode of action may underlie its non-dose-dependent effect. Indeed, many natural products show non-dose-dependent pharmacological activities (24,25).

MAE regulated the levels of 5-hydroxytryptamine and noradrenalin in the serum and hypothalamic of the mice. As a key neurotransmitter in the pathophysiology of migraines, 5-HT is found at low levels in patients with chronic daily headaches (26). Together with other pro-inflammatory mediators, 5-HT reportedly contributes to injury (inflammation)-induced pain (27). Compounds that enhance 5-HT and NE neurotransmission, especially dual-acting antidepressants, may be expected to be effective in the control of chronic pain. In central and peripheral analgesia, the Ca^2+^ channel triggers the K^+^ channel, Cl^-^ channel, and transcription-related genes to realize their physiological functions (28). Our data imply that MAE-mediated analgesic activity is related to the Ca^2+^ channel, but not to opioid receptors, endogenous guanylate cyclase, K^+^ channel, α2-adrenaline, or acetylcholine. Ca^2+^ channel antagonists exert analgesic effects by blocking Ca^2+^ influx and suppressing neurotransmitter release to attenuate impulse conduction in nerve cells (29). According to previous studies, Ca^2+^ channel antagonists can increase the 5-HT and NE levels in the hypothalamus. In contrast, in this study, MAE enhanced the 5-HT and NE levels in the serum, but suppressed their levels in the hypothalami of the mice. Interestingly, tramadol, a weak agonist of synthetic opioid receptors, is recognized as a non-narcotic central analgesic and relieves pain by inhibiting the reuptake of NE and 5-HT in synaptic clefts, blocking calcium influx and decreasing intracellular calcium (30).

Conversely, the activation of 5-HT receptor leads to the opening of receptor-gated ion channels, allowing Ca^2+^ to enter neurons, where it participates in numerous processes such as protein kinase activation (31). CaMKII is well known as an important regulator of calcium signaling in synaptic transmission by phosphorylating various proteins, such as neuronal membrane receptors and intracellular transcription factors (32). CaMKII is a critical regulator of pain signaling and could be a promising downstream target for Ca^2+^/CaMKII-mediated pain sensitization in naive mice (33). INN and MAE significantly inhibited the activation of CaMKII in the hypothalamus after 30-s thermal stimulation. Accordingly, we speculate that co-administration of MAE and Ca^2+^ channel blockers inhibits Ca^2+^ participation in neurons.

In summary, this study demonstrated that MAE exerts significant antinociceptive effects on mice via mechanisms mainly related to Ca^2+^/calmodulin-dependent signaling combined with regulation of monoamine neurotransmitter systems. Our data provide experimental support for the clinical use of *M. androsaceus* mycelium as an analgesic.
